# Upregulation of the PatAB Transporter Confers Fluoroquinolone Resistance to *Streptococcus pseudopneumoniae*

**DOI:** 10.3389/fmicb.2017.02074

**Published:** 2017-10-26

**Authors:** María Alvarado, Antonio J. Martín-Galiano, María J. Ferrándiz, Ángel Zaballos, Adela G. de la Campa

**Affiliations:** ^1^Unidad de Genética Bacteriana, Centro Nacional de Microbiología, Instituto de Salud Carlos III, Madrid, Spain; ^2^Unidad de Genómica, Instituto de Salud Carlos III, Madrid, Spain; ^3^Presidencia, Consejo Superior de Investigaciones Científicas, Madrid, Spain

**Keywords:** DNA topoisomerases, efflux pumps, fluoroquinolones, transcription regulation, *Streptococcus pseudopneumoniae*

## Abstract

We characterized the mechanism of fluoroquinolone-resistance in two isolates of *Streptococcus pseudopneumoniae* having fluoroquinolone-efflux as unique mechanism of resistance. Whole genome sequencing and genetic transformation experiments were performed together with phenotypic determinations of the efflux mechanism. The PatAB pump was identified as responsible for efflux of ciprofloxacin (MIC of 4 μg/ml), ethidium bromide (MICs of 8–16 μg/ml) and acriflavine (MICs of 4–8 μg/ml) in both isolates. These MICs were at least 8-fold lower in the presence of the efflux inhibitor reserpine. Complete genome sequencing indicated that the sequence located between the promoter of the *patAB* operon and the initiation codon of *patA*, which putatively forms an RNA stem-loop structure, may be responsible for the efflux phenotype. RT-qPCR determinations performed on RNAs of cultures treated or not treated with subinhibitory ciprofloxacin concentrations were performed. While no significant changes were observed in wild-type *Streptococcus pneumoniae* R6 strain, increases in transcription were detected in the ciprofloxacin-efflux transformants obtained with DNA from efflux-positive isolates, in the ranges of 1.4 to 3.4-fold (*patA*) and 2.1 to 2.9-fold (*patB*). Ciprofloxacin-induction was related with a lower predicted free energy for the stem-loop structure in the RNA of *S. pseudopneumoniae* isolates (−13.81 and −8.58) than for R6 (−15.32 kcal/mol), which may ease transcription. The presence of these regulatory variations in commensal *S. pseudopneumoniae* isolates, and the possibility of its transfer to *Streptococcus pneumoniae* by genetic transformation, could increase fluoroquinolone resistance in this important pathogen.

## Introduction

*Streptococcus pneumoniae* (the pneumococcus) is a main human pathogen causing community-acquired pneumonia, meningitis, bacteremia, and otitis media (Obaro, 2000). It has been estimated that one million children 5 years of age and under die annually of pneumococcal infections (World Health Organization, 2007). Pneumococcal resistance to antibiotics normally used to treat pneumococcal infections, such as beta-lactams and macrolides has spread worldwide (Liñares et al., 2010). Nowadays, the fluoroquinolones (FQs) levofloxacin (LVX) and moxifloxacin (MXF) are indicated for the treatment of adult patients with pneumonia (Mandell et al., 2007). Although FQ-resistance in *S. pneumoniae* is low (<3%) in Europe (Riedel et al., 2007; Domenech et al., 2014), it is higher in Asia (10.5%, Ip et al., 2007), and in Canada (7.3%, Adam et al., 2009). However, an increase in resistance may occur if the use of these drugs increases (Chen et al., 1999). The intracellular targets of the FQs are the DNA topoisomerase enzymes gyrase and topoisomerase IV, which control DNA topology, solving topological problems associated with replication, transcription and recombination (Champoux, 2001). In *S. pneumoniae*, most FQs act on topoisomerase IV as a primary target (Muñoz and de la Campa, 1996; Morrissey and George, 1999). FQ-resistance in this bacterium occurs mainly by the alteration of their DNA topoisomerases. This can occur either by point mutation or by intraspecific (Stanhope et al., 2005), or interspecific recombination with the genetically related streptococci of the mitis group, commensal microorganisms of the oral cavity (Ferrándiz et al., 2000; Balsalobre et al., 2003; Stanhope et al., 2005). Some FQ-resistant (FQ-R) mutations impose a fitness cost to *S. pneumoniae* (Rozen et al., 2007; Balsalobre and de la Campa, 2008). Compensation of this cost in isolates carrying recombinant topoisomerase genes (Balsalobre et al., 2011) would envisage a future spread of these resistant isolates. These recombinant isolates are originated by genetic interchange with chromosomal DNA of other streptococci of the mitis group. Among them, the species most genetically close to *S. pneumoniae* are *Streptococcus mitis, Streptococcus oralis*, and *Streptococcus pseudopneumoniae* (Arbique et al., 2004). Homologous recombination among streptococci of the mitis group plays a role in the evolution of antibiotic resistance in these bacteria. Recombination had played a role in antibiotic resistance in *S. pneumoniae*. Recombinant genes in the topoisomerase genes (Ferrándiz et al., 2000; Balsalobre et al., 2003), in the genes of penicillin-binding proteins (Dowson et al., 1990) and in the *rpoB* genes of rifampicin-resistant strains (Ferrándiz et al., 2005) have been identified. Recombination and the spread of a few international clones are central factors generating antibiotic resistance in *S. pneumoniae*.

Alternatively, pumping of FQs out of the cell has also a role on resistance in *S. pneumoniae*. FQ-efflux is present in *S. pneumoniae* clinical isolates (Brenwald et al., 1998; de la Campa et al., 2004, 2009) and also in streptococci of the mitis group (Ferrándiz et al., 1999). PmrA, which belongs to the major facilitator superfamily (MFS), was the first pump described in *S. pneumoniae*, whose inactivation in a FQ-R isolate derived from the laboratory strain R6, restored FQ sensitivity (Gill et al., 1999). Further studies pointed out the existence of other FQ-efflux pumps (Pestova et al., 2002; Brenwald et al., 2003). The role of PmrA in FQ-resistance in clinical isolates has not been proved, since no correlation has been observed between *pmrA* expression and FQ-efflux (Piddock et al., 2002). A systematic deletion of 11 putative efflux pumps in *S. pneumoniae* TIGR4 showed an association between deletion of the ABC transporter PatAB and hypersusceptibility to ciprofloxacin (CPX), acriflavine, and ethidium bromide (Robertson et al., 2005). The same phenotype was observed in FQ-R clinical isolates (Garvey et al., 2011). The *patA* and *patB* genes showed CPX-induced overexpression in both a FQ-R laboratory mutant (Marrer et al., 2006), and in clinical isolates (El Garch et al., 2010). Further studies associated mutations in a RNA stem-loop structure located upstream of *patAB* with both the overexpression of these genes and FQ-resistance (Baylay and Piddock, 2015). Likewise, the homolog of PatAB in the zoonotic pathogen *Streptococcus suis*, named SatAB, has been shown to be constitutively overexpressed and to confer CPX resistance to a clinical isolate (Escudero et al., 2011). The *satAB* genes are regulated by the *satR* repressor, which is located upstream of these genes and cotranscribed with them (Escudero et al., 2013). These studies stablished a role for PatAB in FQ-resistance in clinical isolates of *S. pneumoniae*. In addition, a role in LVX and MXF efflux has been stablished for the multi-antimicrobial extrusion family (MATE) DinF transporter in a pneumococcal laboratory strain (Tocci et al., 2013).

Among the FQ-R (considering those with CPX MICs ≥4 μg/ml) *S. pneumoniae* isolates characterized in our laboratory in two epidemiological studies, one performed during 1991 to 2001 (Ferrándiz et al., 2000; Balsalobre et al., 2003), and the other in 2002 (de la Campa et al., 2004), we selected 14 isolates carrying DNA topoisomerase genes with high nucleotide sequence variations (>4%). Seven out of these 14 isolates were further classified as *S. pseudoneumoniae* (Balsalobre et al., 2013). Two of them, 5305 and CipR-71, which did not carry mutations in the quinolone-resistance determining regions of their topoisomerase genes, showed efflux as a single mechanism of FQ resistance. As far as we know, there are no other descriptions of FQ-resistant *S. pseudoneumoniae*. In this study we aimed to identify the efflux mechanisms of FQ-resistance of these isolates. Whole genome sequencing and genetic transformation experiments were performed together with phenotypic determination of FQ-efflux.

## Materials and methods

### Bacterial strains and conditions for growth and transformation

*Streptococcus pneumoniae* and *Streptococcus pseudopneumoniae* strains were grown as static cultures either in a casein hydrolysate based medium (AGCH) supplemented with 0.3% sucrose and 0.2% yeast extract (A+SY) (Lacks et al., 1986) or in Tod-Hewitt (Becton Dickinson) with 0.5% of yeast extract (THY). MICs of ethidium bromide, acriflavine and FQs were determined by agar-dilution (for *S. pseudopneumoniae* 5305) or by macrodilution following the methods of the CLSI (Clinical Laboratory Standards Institute, 2008) using 2-fold dilutions in A+SY liquid medium, with an inoculum of 5 × 10^5^ cells per well. The efflux inhibitor reserpine (Gill et al., 1999; Garvey et al., 2011) was used at a final concentration of 10 μg/ml, which has been shown to inhibit ethidium bromide, acriflavine, and CPX efflux in streptococci of the mitis group (Ferrándiz et al., 1999). *S. pneumoniae* R6 was used as the recipient in transformation experiments. Competent cultures containing 9 × 10^6^ CFU/ ml were incubated with 0.1 μg/ ml of DNA during 40 min at 30°C and then at 37°C for 90 min before plating. Colonies were counted after 24 h of growth at 37°C in a 5% CO_2_ atmosphere in A+SY media plates containing 1% agar and CPX at 2 μg/ ml. No transformation refers to experiments in which no colonies were observed when 100 μl of the transformation mixture was plated, which gave a frequency <1 × 10^1^. DNA used as control was the chromosome of T5305 that yielded 1.1 × 10^3^ transformants/ml.

### Whole-genome sequencing

Two *S. pseudopneumoniae* clinical isolates, 5305 and CipR71, and two *S. pneumoniae* strains, an R6 transformant with 5305 chromosomal DNA, named *S. pneumoniae* T5305 and our laboratory *S. pneumoniae* R6 strain, were subjected to whole genome sequencing. Genomic DNA samples were prepared from mid-log phase cultures using the CTAB protocol (Wilson, 1994). Sequencing was performed either in a 454 Life Sciences GS-FLX+ system, Roche (5305 and CipR71) or in an Illumina NextSeq 500 system (T5305 and our laboratory R6). DNA for 454/Roche library preparation was fragmented by nebulization and posterior quantification, emulsion PCR and sequencing performed as recommended by the manufacturer. Libraries for Illumina sequencing were obtained following the transposon-mediated Nextera XT library preparation kit (Illumina) and paired-end sequenced with a 2 × 150 protocol. De novo or reference aided (*S. pneumoniae* R6) assemblies were performed using Newbler 3.0 (Roche) with default settings. sff files from 454/Roche and fastq files from Illumina were used as input sequences, respectively. Assembly metrics are shown in Table 1. Coverage was calculated by dividing total bases used in the computation by the size of the final alignment following the Lander/Waterman equation (Lander and Waterman, 1988).

**Table 1:** Characteristics of the whole genome sequences.

**Strain**	**Total reads^b^**	**Contig size**			**Mean coverage**
		**N°**	**N° bases**	**N50^c^**	
*S. pseudopeumoniae* 5305	89,125	129	2,160,666	44,063	21
*S. pseudopeumoniae* CipR71	79,852	101	2,141,171	74,053	20
*S. pneumoniae* T5305	2,809,086	221	1,976,262	32,462	181
*S. pneumoniae* R6^a^	1,450,983	52	2,007,471	91,805	84

a *Our laboratory S. pneumoniae R6 strain*.

b *The % of aligned reads was ≥98.60 and of the aligned bases was ≥97.56*.

c *Minimum contig length needed to cover 50% of the genome*.

### Gene and protein analysis

Coding sequences were predicted by Prodigal V.2.6.3 with default settings (Hyatt et al., 2010). *S. pneumoniae* pump gene sequences (Tocci et al., 2013) were detected in *S. pseudopneumoniae* genomes by BLASTN using *S. pneumoniae* TIGR4 sequences as queries and applying bidirectional thresholds of ≥80% identity and an alignment length over ≥80% of the protein sequences. Promoter-containing sequences of *S. pseudopneumoniae* isolates were extracted as the 100 upstream nucleotides of the pump gene, or, in the case of genes in operons, the 100 upstream nucleotides of the first gene of the operon. The RNA stem-loop structure was predicted by using the RNA-fold web server (http://rna.tbi.univie.ac.at/cgi-bin/RNAWebSuite/RNAfold.cgi). Gene organization of operons was downloaded from the DOOR database (Mao et al., 2009), using the *S. pseudoneumoniae* IS7493 strain as reference. Protein and nucleotide sequences were aligned with Clustal Omega (Sievers et al., 2011).

### Nucleotide sequence accession numbers

The sequencing data have been deposited at the whole genome sequencing database linked to the BioProject ID PRJNA386436.

### PCRs, RNA extraction and quantitative real time PCR (RT-qPCR) procedures

Primers used for PCR amplifications and sequencing are shown in Table 2. Platinum® Taq High Fidelity (Invitrogen), was used for PCR amplifications in the following conditions: an initial cycle of 2 min denaturation at 94°C; 30 cycles of denaturation at 94°C for 30 s, annealing at 55°C for 30 s and extension at 68°C for 1 min per kb of PCR product. Primers used for PCR amplifications and sequencing are shown in Table 2. To quantify the relative expression of *hexA, patA*, and *patB*, a procedure previously described (El Garch et al., 2010) was used. Briefly, R6-derivative strains were grown overnight at 37°C in 5% CO_2_ in Mueller-Hinton agar plates supplemented with 5% defibrinated sheep blood (Becton Dickinson). Bacteria were suspended in 5 ml of THY, grown to OD_620nm_ = 0.2, diluted 10-fold into 25 ml of THY and grown to OD_620nm_ = 0.2 to 0.4 (2–4 × 10^8^ cells). At this point, cultures were split into two parts; one part was grown during 4 h in the presence of CPX at 0.5 × MIC for each strain, and the other was grown in its absence. Cells were collected by centrifugation and their total RNA was extracted with an RNeasy mini kit (Qiagen), with 3 DNase I (Qiagen) treatments, according to the manufacturer's instructions. Synthesis of cDNAs were carried out in 20-μl reactions containing 0.5–5 μg of RNA, 0.5 mM of each deoxynucleoside triphosphate, 2 pmol of hexanucleotide random primers, 40 U of the RNaseOUT RNase inhibitor (Invitrogen), 200 U of SuperScript IV reverse transcriptase (Invitrogen), and 2 U of RNase H^−^ (Invitrogen), in the buffer recommended by the manufacturer. These cDNAs were used in RT-qPCR amplifications (Light cycler 480, Roche). The total volume in each reaction was 20 μl and contained 2 μl of cDNA, 0.4 μM of each primer, and 10 μl of Sso Advanced Universal SYB Green Supermix (BioRad). The program had one initial denaturation cycle of 4 min at 94°C followed by 42 cycles of three steps: denaturation (30 s at 94°C), annealing (30 s at 54°C), and elongation (30 s at 68°C). Oligonucleotide pairs used were: PatARTF/PatARTR (*patA*); PatBRTF/ PatBRTR (*patB*), HexARTF/HexARTR (*hexA*). The amount the RT-PCR products was calculated with a standard curve for each amplicon. Assays were performed in triplicate and normalized against the signal from a 16S rDNA internal control (oligonucleotide pair rDNAF/rDNAR).

**Table 2:** Oligonucleotides used in this work.

**Name**	**Sequence (5′-3′)^a^**	**Nucleotide positons^b^**
HexAF22	GGCATGCAACAGTATGTGG	64 to 82 of *hexA*
HexAF757	CCAAGATTGCTGGCTTGCCAGC	2,330 to 2,352 of *hexA*
HexAR755	CCAGCAATCTTGGCAACATGG	Complementary to 2,342 to 2,321 of h*exA*
HexARTF	GCTCTTTCGGATGGGTGATTT	113 to 134 of *hexA*
HexARTR	GCTTACTGCTATCGACCACTGT	Complementary to 378 to 400 of *hexA*
HexARTF2	CATCCTATCCTAGCAGAATTAGC	2,479 to 2,501 of *hexA*
HexARDown	TTATAGTTTCTGTTTTAACTCTAC	Complementary to 2,551 to 2,574 of *hexA*
GuaR80	GGATGAACATTTTGGCCTCCTG	Complementary to 474 to 452 of *guaA*
PatAF17	GATGACAGGCTTGATGGTTGC	53 to 74 of *patA*
PatAR17	GCAACCATCAAGCCTGTCATC	74 to 53 of *patA*
PatBFUp	CGCATGCAGACTTGGTTGCCA	−827 to −806 of *patB*
PatARDown	TGGCAACCAAGTCTGCATGCG	Complementary to −806 to −827 of *patB*
PatBRDown	TAGGACAAGAAAAAGCAGCCCC	Complementary to −73 to −51 of *guaA*
PatARTF	TGATGACAGGCTTGATGGTT	52 to 72 of *patA*
PatARTR	AACGACTAGATTTCCCGCAT	Complementary to 333 to 313 of *patA*
PatARTR2	AACCATCAAGCCTGTCATCA	Complementary to 53 to 72 of *patA*
PatARACE1	CTCCTTGGGCAATATAGGCTGC	Complementary to 220 to 241 of *patA*
PatARACE2	CCAATCAACCAAGCCCCGATAC	Complementary to 152 to 173 of *patA*
PatBRTF	GCACCCCATTGGCTTTCCTTA	523 to 542 of *patB*
PatBRTR	GCACGCGCTCATTTTGTTCA	Complementary to 715 to 695 of *patB*
rDNAF	GGTGAGTAACGCGTAGGTAA	101 a 120 of 16SrDNA
rDNAR	ACGATCCGAAAACCTTCTTC	Complementary to 407 to 426 of 16SrDNA
RibAF135	GCTTCTGTGTGTCCATTTCTTTC	411 to 434 of *ribA*
RACE 5′	GATATGCGCGAATTCCTGTAG	1 to 21 of RNA adaptor
RACE5′Inner	AATTCCTGTAGAACGAACACTAGAA	11 to 35 of RNA adaptor
RNA adaptor	GAUAUGCGCGAAUUCCUGUAGAACGAACACUrArGrArArArGrArArA	
TagAR131	GGGGCTTGGCGATAATCAGG	Complementary to 413 to 393 of *tagA*

a *The “r” before the base indicated that it was a ribonucleotide*.

b *Nucleotide and amino acid numbering refers to the genes and proteins obtained from the S. pneumoniae R6 sequence, with the first nucleotide or amino acid at position 1*.

### Transcription start site determination

5′ RACE (rapid amplification of cDNA ends) method was performed using total RNA extracted from strain TCipR71.2 treated with CIP at 0.5 × MIC as template. A total of 12 μg of RNA were used, 6 μg were treated with Tobacco acid pyrophosphatase (TAP) to hydrolyze specifically 5′-triphosphate from primary transcripts, and to subsequently be ligated to the 3′-hydroxyl group of an RNA oligonucleotide (RNA adaptor, Table 2). The remaining 6 μg of total RNA were not treated with TAP and followed the same post-treatment. cDNAs were synthesized from these RNAs using SuperScript IV Reverse Transcriptase (Invitrogen) following manufacturer recommendations and used as templates in PCR reactions with primers PatARACE1 and RACE5′ (Table 2) as detailed in PCRs, RNA Extraction and Quantitative Real Time PCR (RT-qPCR) Procedures. PCR products were purified with MinElute PCR purification kit (Quiagen) and used as templates for a second PCR reaction using primers PatARACE2 and RACE5′Inner (Table 2). PCR products were separated in a 2% Metaphor (FMC Bioproducts) agarose gel and the band that appeared in the sample treated with TAP, and did not appear in the untreated sample, was isolated from the gel with MinElute Gel Extraction Kit (Qiagen). DNA was sequenced using primer PatARACE2 to determine the transcription start site, which was the first base 5′ immediately after the adaptor sequence.

## Results

### Efflux phenotypes of *S. pseudopneumoniae* isolates

The MICs of isolates 5305 and CipR71 to FQs and some dyes in the presence and absence of the efflux pump inhibitor reserpine were compared to three efflux-negative control strains: *S. pneumoniae* R6, *S. pseudopneumoniae* CCUG48465, and the *S. pseudopneumoniae* type strain CCUG49455^T^. No significant differences in the MICs of LVX, CPX, ethidium bromide and acriflavine in the presence or absence of reserpine were observed in the control strains. However, the two *S. pseudopneumoniae* clinical isolates, which showed 8- to 32-fold higher MICs of these drugs in the absence of reserpine than the control strains, essentially behave like the control strains in the presence of the pump inhibitor (Table 3). Reserpine it is assumed not to affect growth of the strains at the concentration used (10 μg/ ml), since the MICs of reserpine for *S. pneumoniae* have been described to be 60 μg/ml (Garvey and Piddock, 2008). These results suggested the existence of an efflux pump in *S. pseudopneumoniae* isolates 5305 and CipR71.

**Table 3:** Susceptibilities of strains to fluoroquinolones and efflux pump substrates.

**Strain**	**MIC (**μ**g/ml)**^a^							
	**LVX**	**LVX+Rs**	**CPX**	**CPX+Rs**	**EtBr**	**EtBr +Rs**	**Acr**	**Acr+Rs**
*S. pneumonia* R6	0.25	0.25	0.5	0.5	1	0.25	1	0.5
*S. pseudopneumoniae* CCUG49455^T^	0.25	0.25	0.5	0.25	0.5	0.25	0.5	0.25
*S. pseudopneumoniae* CCUG48465	0.5	0.5	0.5	0.25	0.5	0.25	0.5	0.25
*S. pseudopneumoniae* 5305	1	0.5	**4**	0.5	**16**	0.25	**8**	1
*S. pneumonia* T5305	1	0.5	**4**	0.25	**16**	0.25	**4**	1
*S. pneumonia* T5305.1	1	0.5	**4**	0.25	**16**	0.25	**8**	1
*S. pneumonia* T5305.2	1	0.5	**4**	0.25	**16**	0.25	**4**	1
*S. pseudopneumoniae* CipR71	1	0.5	**4**	0.25	**8**	0.25	**4**	1
*S. pneumonia* TCipR71.2	1	0.5	**4**	0.25	**16**	0.25	**4**	1

a *Acr, acriflavine; CPX, ciprofloxacin; EtBr, ethidium bromide; LVX, levofloxacin; Rs, reserpine. MICs are the average of at least three determinations, which did not differ more that 2-fold dilution. Figures in bold indicate MICs at least 2 dilutions higher in the absence of reserpine than those of R6 and the S. pseudopneumoniae CCUG48465, and S. pseudopneumoniae type strain CCUG49455^T^*.

### Genome sequence of *S. pseudopneumoniae* ciprofloxacin-resistant strains

We analyzed the genome sequences of the 5305 and CipR71 resistant strains and compared them with those of *S. pneumoniae* and *S. pseudopneumoniae* isolates, paying special attention to those coding for the 11 pneumococcal antimicrobial pumps (Tocci et al., 2013). *S. pseudopneumoniae* isolates carried nine of these pumps, coded by 14 genes, given that they are constituted by complexes in some cases. Amino acid sequences of the permease subunits and the sequences covering the 100 nucleotides upstream of the coding sequence (or of the first coding sequence of the operon) were compared (Table 4). Subtle differences were detected in the sequences of these pumps. However, simultaneous alterations, i.e., alterations common to both *S. pseudopneumoniae* isolates with an efflux phenotype (CipR71 and 5305) respect to the efflux-negative *S. pseudopneumoniae* type strain ATCC BAA-960, were found only in the coding sequences of five of these genes (affecting four pumps), which essentially ruled out the possible involvement of the nine remaining genes in the efflux phenotype. The most remarkable efflux-linked determinants were the coding sequence of the ABC transporter SP_1357 (11 residue changes) and the regulatory sequence of the *patAB* operon. Both proteins are part of the Drug Exporter-4 family of the Transport Classification Database (TCDB id: 3.A.1.135). The enhanced CPX efflux of isolates 5305 and CipR71 could be attributed either to higher substrate affinity/enhanced activity produced by the residue changes in SP_1357, by an up-regulation of the PatAB pump, or by a combination of both. To discern between these possibilities, genetic transformation experiments were carried out as described below.

**Table 4:** Comparison of coding and regulatory sequences of *S. pseudopneumoniae* homologs to *S. pneumoniae* pumps associated to drug efflux.

**Operon or gene^a^**	**Transporter^b^**	**Coding sequence**^c^				**Upstream sequence**^d^		
		**Gene**	**% Identity**		**N° of simultaneous changes**	**%Identity (indels)**		**N° of simultaneous SNPs**
			5305	CipR71		5305	CipR71	
SP0967-72	MFS-PmrA	SP0972	97.3^e^	100^f^	0	100 (0)	100 (0)	0
SP1116	ABC	SP1116	95.9	100	0	96 (0)	98 (0)	0
SP1166	MATE	SP1166	98.9	98.2	1	100 (0)	100 (0)	0
SP1359-7	ABC-MDR1	SP1358	98.3	100	0	95 (0)	98 (0)	0
		SP1357	96.6	98.1	11	–	–	–
		SP1840	100	100	0	–	–	–
SP1840-39	ABC-MDR1	SP1839	99.5	100	0	100 (0)	100 (0)	0
SP1919-8	ABC	SP1919	98.4	99.3	2	96 (0)	97 (0)	0
		SP1939	96.1	100	0	–	–	–
SP1939	MATE-DinF	SP2065	95.8	100	0	100 (0)	100 (0)	0
SP2065	MATE	SP2075	98.9	99.2	2	95 (0)	100 (0)	0
SP2075-3	ABC-MDR1-PatAB	SP2073	98.0	99.3	2	94 (1)	95 (1)	3

a *Loci of TIGR4 (GenBank accession number AE005672), operon indicates the genes included in the same transcriptional unit, as loaded from the DOOR database (39)*.

b *ATP binding cassette (ABC); multiantimicrobial extrusion (MATE); major facilitator superfamily (MFS)*.

c *Identities (%) in the coding sequence (amino acid residues) of the permease subunits with respect the S. pseudopneumoniae type strain ATCC BAA-960*.

d *The sequences positions −101 to −1 of the gene/ first gene of the operon were compared. −, no applicable*.

e *S. pseudopneumoniae 5305 has 1-30 and 325-393 deletions for this protein*.

f *S. pseudopneumoniae CipR71 has a 80 residues insertion in the C-terminus (394-473)*.

### Genome sequence of the R6 transformant (T5305) revealed two recombination regions

**Figure 1:**
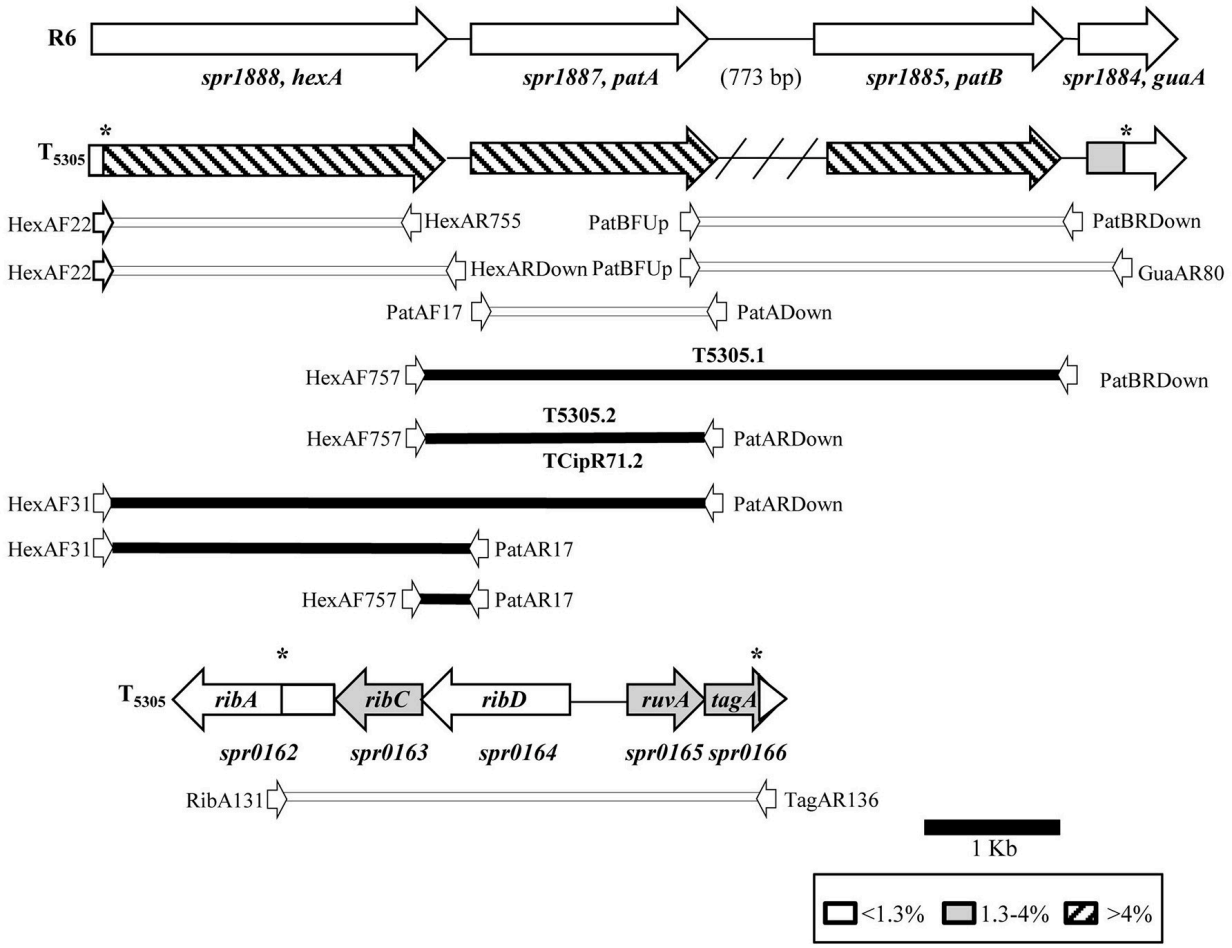
Genomic map of the recombination regions of *S. pneumoniae* T5305. Genes are represented as arrows, indicating the level of nt divergence with respect SPN R6. PCR fragments used in transformation experiments, together with their corresponding primers (white arrows, not draw to scale), are indicated. The intergenic region between *patA* and *patB* is 773-bp in *S. pneumoniae*, but is absent in *S. pseudopneumoniae* isolates (indicated by a crosshatched line). Black boxes and white boxes indicate PCR fragments yielding or not yielding CPX-R transformants, respectively. Asterisks indicate recombination points.

*Streptococcus pneumoniae* R6 was transformed with chromosomal DNA of *S. pseudopneumoniae* 5305. One transformant, T5305, was selected. T5305 had a CPX MIC of 4 μg/ml, this MIC was equivalent to that of *S. pseudopneumoniae* 5305. In addition, 5305 and T5305 exhibited the same efflux phenotype to CPX, ethidium bromide and acriflavine (Table 3). The analysis of the *S. pneumoniae* T5305 genomic sequence showed 420 nucleotide changes with respect to the sequence of *S. pneumoniae* R6 present in the NCBI data base (Accession number: NC_003098.1). To discard spurious mutations, our laboratory R6 strain was also sequenced, showing 21 SNPs with respect to the NCBI sequence (data not shown). These changes were not considered, then, a total of 399 changes were attributed to the recombination event. These changes were unevenly distributed, 353 of them were located in a 7184 bp region (4.8% of variation) covering the spr1884 to spr1888 genes (*guaA*-*patB*-*patA*-*hexA*); 45 in a 3540 bp region (1.2% of variation) covering the spr0162 to spr0166 genes (*ribA*-*ribC*-*ruvA*-*tag*); and 1 point mutation in spr1903 (*galU*). These results demonstrate that the recombination event yielding T5305 occurred in two regions: spr0162-0166 and spr1884-1888 (Figure 1). This was very likely due to its close spatial proximity considering both recombined regions are at the same distance to the chromosome replication origin, a factor that may increment inter-replicore gene transference.

### The region involved in FQ efflux includes the upstream *pata* region

**Figure 2:**
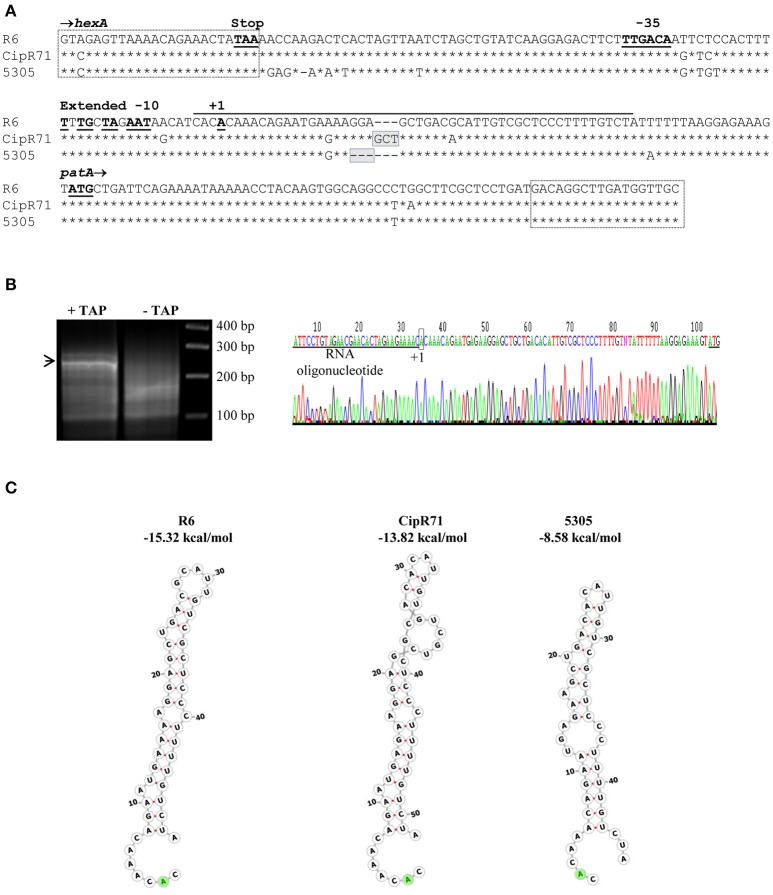
Sequence of the region involved in CPX resistance in *S. pseudopneumoniae* isolates, determination of the *patAB* transcription star site, and putative structures in the *patA* upstream RNAs. **(A)** Sequence included between oligonucleotides HexARDown and PatAR17, which are boxed, is shown in full for *S. pneumoniae* R6. For *S. pseudopneumoniae* strains CipR71 and 5305, only nucleotide changes are indicated, with nucleotides identical to R6 indicated by asterisks, and deletions by dashes. **(B)** 5′ RACE PCR method. Agarose gel electrophoresis of PCR products of samples treated (+) or non-treated (–) with tobacco acid pyrophosphatase (TAP). The band indicated by an arrowhead was extracted and sequenced. The chromatogram, sequence of the RNA oligonucleotide, star site, and initiation codon of *patA* are indicated. **(C)** Structures of the regions marked with a black line in **(A)** as predicted by RNA-fold. The A shadowed in green indicates the start site.

To reveal the role of the nucleotide changes present in T5305 in FQ-efflux, diverse DNA fragments were amplified from this strain by PCR with specific oligonucleotides (Table 2). These fragments were used as donor DNAs in transformation experiments with R6 as receptor, and transformants selected at 2 μg/ml of CPX. Transformation experiments were performed as described under Materials and Methods, being the frequencies of transformation in the range of 1 to 7 × 10^3^ transformants/ ml. DNA fragments covering the *spr*0162-0166 genes or the *galU* mutation did not result in transformants at elevated CPX concentrations, discarding the role of these genes in CPX-resistance. In contrast, two overlapping PCR fragments from the *spr*1884-1888 region transformed R6 with high efficiency (Figure 1). One of the amplicons covers the C-terminus of *hexA* and the intergenic *hexA*-*patA* region plus *patA*, whereas the other covers the same regions but including *patB*. In accordance, PCR products including most of the *hexA* and *patA* sequences were also able to transform to CPX resistance. The smallest fragment able to transform R6 to CPX resistance was obtained by amplification with oligonucleotides HexAF757 and PatAR17. Likewise, PCR products obtained from isolate CipR71 with the same oligonucleotide pairs also transformed R6 with high efficiency. In contrast, DNA fragments containing *hexA, patA, patB*, or *guaA* coding sequences, but not the *hexA*-*patA* intergenic region, were unable to transform R6 to CPX-resistance. In addition, a fragment covering *hexA* from residue position 22 to the stop codon, which was obtained from *S. pseudopneumoniae* strains 5305 and CipR71, did not transform to CPX-resistance (Figure 1). Three resistant transformants, two obtained with PCR products from 5305 (T5305.1 and T5305.2) and one from CipR71 (TCipR71.2) were selected for further studies. Their nucleotide sequences were identical to that of the donor 5305 and CipR71 strains and their MICs were equivalent to that of their parental DNA donors (Table 3). All these results demonstrated that the region involved in FQ-efflux in the *S. pseudopneumoniae* strains T5305 and CipR71 was that included between oligonucleotides HexARDown and PatAR17 (Figure 2A). This region contains the intergenic *hexA*-*patA* region and the first 74 nucleotides of *patA*. No amino acid changes were predicted in PatA between R6, 5305 and CipR71 or the derived R6-transformants. The intergenic *hexA*- *patA* region has been previously stablished to be involved in FQ-efflux in SPN (Baylay and Piddock, 2015). A promoter with a canonical −35 box and an extended −10 sequence was observed in all strains. We determined the transcription start site by RACE 5′ PCR as described in materials and methods, which was located eight nucleotides downstream of extended −10 sequence (Figure 2B). The main differences were found between the extended −10 sequence and the *patA* initiation codon, in which the two *S. pseudopneumoniae* FQ-resistant strains CipR71 and 5305 showed a 3-nt insertion (GCT) or a 3-nt deletion (GGA), respectively. A stem-loop structure could be predicted by RNA-fold in this region with a free energy of −15.32 kcal/mol for R6 (Figure 2C). The nucleotide changes observed in CipR71 and 5305 CPX-resistant isolates are predicted to lower the free energies of these structures to −8.58 kcal/mol for 5305 and −13.82 kcal/mol for CipR71. These changes in the stability of the stem-loop structures could cause alterations in the transcription of the downstream *patA* and/ or *patB* genes.

### Transcription of *patA* is induced by CPX

**Figure 3:**
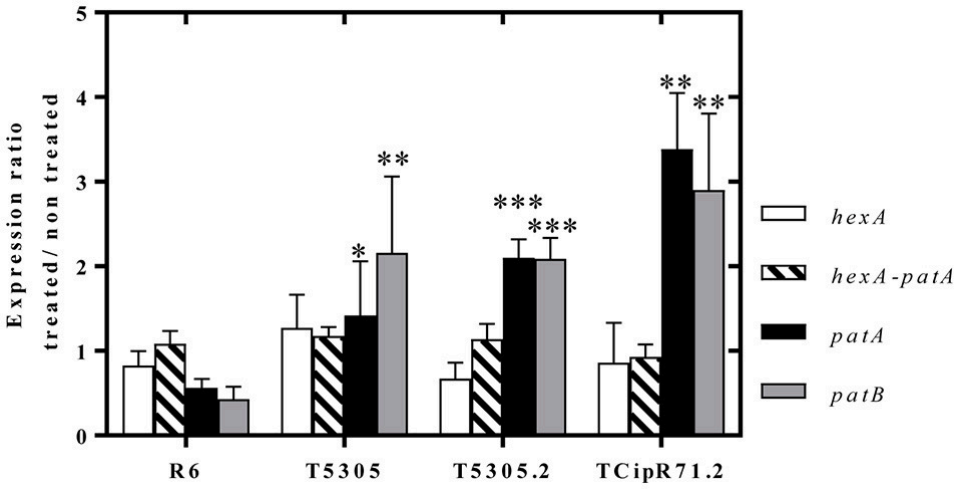
Expression of *hex*A, *pat*A and *pat*B in R6-derivative *S. pneumoniae* strains cultures exposed for 4 h to 0.5 × MIC of CPX. Data are represented as ratios of expression under treated and non-treated cultures. Values are mean ±SD of triplicates from three independent experiments. Statistical significance with respect to R6 values by Student *T*-test: ^*^P ≤ 0.05; ^**^P ≤ 0.01; ^***^P ≤ 0.001.

To quantify the induction produced by CPX, RT-qPCR experiments were performed with RNAs of cultures treated or not treated with a subinhibitory CPX concentration. These assays showed that the expression of both *patA* and *patB* in the R6-derivative strains was induced by CPX. While no significant changes were observed in R6, increases in *patA* and *patB* were detected in all strains with an efflux phenotype. These increases were in the range of 1.4 to 3.4-fold for *patA*, and of 2.1 to 2.9-fold for *patB*. However, the transcription of *hexA* did not show significant differences between R6 and its transformant-derived strains, being in the range of 0.7 to 1.3-fold (Figure 3).

## Discussion

In this study we have characterized the FQ-efflux phenotype of two *S. pseudopneumoniae* isolates, 5305 and CipR-71, which do not have mutations in their DNA topoisomerase genes. Changes in the regulatory sequences of the *patAB* operon were found. The presence of these changes in two genetically diverse isolates is significant. CipR71 is genetically closer to the *S. pseudopneumoniae* reference strain than to 5305, as inferred from the higher identity between pump sequences (Table 4) and the fact they share three out of seven alleles (*ddl, gki* and *recP*) used in multilocus sequence typing, whereas 5305 shares none. These changes would have been acquired by horizontal transfer and/or convergent evolution processes. In addition, these sequence features were absent or very unusual in *S. pseudopneumoniae* as corroborated in the only complete genome fully sequenced in this species—IS7493—and the 29 *S. pseudopneumoniae* strains present in the whole-genome shotgun contig database of the NCBI that were screened. In addition, other pumps, whose function has not been determined, were detected in the CPX-efflux isolates (data not shown).

The analysis of the T5305 sequence, together with transformation experiments, showed that the region involved in CPX-efflux, which has the greatest variation by far with respect to R6, was a 7184 bp region covering *spr*1884 to *spr*1888 genes (*guaA*-*patB*-*patA*-*hexA*). The region involved was further reduced to 247-bp, independently of the donor DNA strain (5305 or CipR71). This region contains the last 24 nt of *hexA*, the intergenic *hexA*-*patA* region, and the first 74 nt of *patA*. No amino acid changes were predicted in HexA or PatA between R6, 5305 and CipR71, and therefore we conclude that the intergenic *hexA*- *patA* region previously established to be involved in FQ-efflux in *S. pneumoniae* (Baylay and Piddock, 2015), is also involved in *S. pseudoneumoniae*. Then, the enhanced CPX efflux of isolates 5305 and CipR71 could be attributed to the regulation of the expression of the *patAB* operon, and not to residue changes in the proteins of the pump. The main difference was found in the sequence between the extended−10 region and the *patA* initiation codon, in which a 3-nt insertion (GCT) in the case of CipR71, or a 3-nt deletion in the case of 5305, were observed. These indels were not found in the *patAB* upstream regions of the reference genome or in a set of *S. pseudoneumoniae* genomes in the whole-genome shotgun database. Since this genome set include diverse isolates from disparate geographical origins and sample sources, these indels—and subsequently the efflux phenotype that provides—can be considered as an unusual feature selected by the pressure exerted by the presence of FQs. These indels would affect the stability of the stem-loop structure located downstream of the −10 extended box of the promoter and the mRNA start site. In fact, the predicted free energies for this stem-loop structure were lower for *S. pseudopneumoniae* isolates showing FQ efflux than for R6. The presence of these alternative structures may influence the amount of the *patAB* transcript under CPX-induction, as showed by RT-qPCR. Differences between 5305 PatAB and *S. suis* SatAB protein sequences were actually observed (84–86% similarity), where non-conservative changes are concentrated in the two first transmembrane alpha-helices (data not shown). This variability may account for subtle differences in the FQ specificity of homologs of this protein family potentially found in other streptococci.

The genome of *S. pneumoniae* (de la Campa et al., 2017) and of other streptococci (Martín-Galiano et al., 2017) is organized in supercoiling domains that react coordinately to changes in supercoiling. Transcriptomic studies of *S. pneumoniae* R6 showed that *patA* and *patB* are up-regulated in response to DNA relaxation with novobiocin, an inhibitor of DNA gyrase (Ferrándiz et al., 2010) and also by hyper-negative supercoiling resulting from the inhibition of DNA topoisomerase I by seconeolitsine (Ferrándiz et al., 2016a). Then, *patA* and *patB* genes are located in chromosomal domains that are up-regulated by global changes in DNA supercoiling. This fact may reflect the high functional weight of this pump under threatening conditions for the chromosome supercoiling state caused by xenobiotics, such as FQs. Both ethidium bromide and acriflavine, two substrates of the PatAB pump, are DNA intercalants that alter the supercoiling of the DNA, causing DNA relaxation (Ladoulis and Gill, 1970; Funatsuki et al., 1997). In addition, acriflavine has been shown to inhibit the DNA binding of gyrase (Mraheil et al., 2013). The effect of the treatment with either of these compounds would cause DNA relaxation, and, as in the case of inhibition of gyrase B with novobiocin, the transcription of the *patAB* operon would be enhanced. Although FQs do not change global supercoiling in *S. pneumoniae*, local supercoiling changes have been shown to alter the transcriptome of cultures treated with either LVX (Ferrándiz and de la Campa, 2014) or MXF (Ferrándiz et al., 2016b). Transcriptomic changes were different with these two different FQs, but, in no case, the transcription of *patAB* was affected. However, the transcriptional response to CPX, which also differed from that of LVX or MOX, involves the up-regulation of *patAB* (Marrer et al., 2006). We proposse that local changes in DNA supercoiling would affect the stability of the stem-loop structure located upstream *patA* (Glucksmann et al., 1992; Lionnet et al., 2006), regulating the amount of the transcripts of the downstream genes, either by regulation of transcription or by processing of the mRNA. The role of a transcriptional regulator recognizing the hairpin would be compatible with this hypothesis.

The mutations described in this study did not confer clinical FQ resistance by itself, since only conferred resistance to CPX at 4 μg/ml, in agreement with previous studies (Zhanel et al., 2004; El Garch et al., 2010; Lupien et al., 2013). However, it has been described that the presence of CPX efflux pumps favors the acquisition of further mutations in the topoisomerase genes, and that the combination of efflux and first-step mutations can lead to MICs associated with treatment failure (Jumbe et al., 2006). Then, the genetically close species *S. pneumoniae* and *S. pseudopneumoniae*, shared the same mechanisms of FQ resistance. Besides mutations in the quinolone-determining regions of their topoisomerase genes, they also shared the up-regulation of the PatAB pump as a mechanism of FQ-efflux. In the same way as mutations in the topoisomerase genes can be transferred between commensal streptococci of the mitis group and *S. pneumoniae* (Ferrándiz et al., 2000; Balsalobre et al., 2003), the mutations in the regulatory region of *patA* could be exchanged, as shown in this study, and could therefore contribute to a future increase of FQ resistance in *S. pneumoniae*. These results reinforce the notion that *S. pseudopneumoniae* can turn into an exchanger of resistance determinants to *S. pneumoniae*, since both organisms share ecological niche (they inhabit for long-term in the nasopharynx, Sakwinska et al., 2014), and show high nucleotide identity and sinteny of flanking regions, which would favor horizontal transfer of their genetic material.

## Author contributions

MA and MF performed the experimental work. AM and AZ analyzed genome sequences. AdlC participated in study conception, data interpretation and manuscript writing. All authors participated in data interpretation and manuscript corrections.

### Conflict of interest statement

The authors declare that the research was conducted in the absence of any commercial or financial relationships that could be construed as a potential conflict of interest.
